# Newborn IgG and IgM antibodies to maternal SARS-CoV-2 infection in pregnancy and child neurodevelopmental outcomes

**DOI:** 10.1016/j.bbi.2026.106591

**Published:** 2026-04-11

**Authors:** Lisa A Croen, Yinge Qian, Stacey Alexeeff, Jennifer L. Ames, Paul Ashwood, Luke Grosvenor, Danielle HJ Kim, Judy Van de Water, Robert Yolken

**Affiliations:** a Division of Research, Kaiser Permanente Northern California, Pleasanton, CA, United States; b Kaiser Permanente School of Medicine, Pasadena, CA, United States; c University of California at Davis, Davis, CA, United States; d Department of Pediatrics, Johns Hopkins School of Medicine, Baltimore, MD, United States

**Keywords:** COVID-19, Maternal infection, Pregnancy, Autism, Neurodevelopmental disorders, Antibodies

## Abstract

Maternal immune activation is a hypothesized mechanism by which prenatal exposure to infection adversely impacts fetal brain development. SARS-CoV-2 IgG class antibodies generated to viral proteins in pregnancy are transferred across the placenta to the newborn.

This study aimed to investigate the association between neonatal levels of IgG antibodies to SARS-CoV-2 nucleocapsid and spike proteins and risk of neurodevelopmental disorders (ND) among children with gestational SARS-CoV-2 exposure. Using a longitudinal prospective cohort design within an integrated healthcare system with a diverse patient population in Northern California, the study looked at 399 children born to mothers who had SARS-CoV-2 infection during pregnancy and had not received the COVID-19 vaccination. Exposure was IgG and IgM class antibodies to SARS-CoV-2 nucleocapsid, IgG directed against the full-length spike protein, the s1-receptor binding domain (RBD) and s1 N-terminal domain (NTD) of the spike protein, and IgM to the full length and RBD spike proteins. The main outcome and measure were all ND, including autism spectrum disorder (ASD), speech/language delay, and motor delay. Cox proportional hazards regression models estimated hazard ratios (HR) and 95% confidence intervals (CIs), with adjustment for maternal sociodemographic and clinical characteristics, and child sex. Among the 399 study children (208 females,191 males) followed up to 64 months, 95% had gestational age ≥ 37 weeks and birthweight ≥ 2500 g. The mean maternal age was 31 +− 5 years; 12% were Asian, 5% Black, 45% Hispanic, 33% White, and 5% other/unknown race/ethnicity; 80% had more than a High School education; 86% had commercial insurance; 37% were primiparous; and 35% had pre-pregnancy obesity. A total of 73 children were diagnosed with ND in follow-up (22 ASD, 67 speech/language disorder, 11 motor delay). Associations between SARS-CoV-2 nucleocapsid IgG antibody level and risk of ND differed significantly by child sex, with increased risk observed only among males (ND: aHR = 1.25 [1.01–1.56]; ASD: aHR = 1.38 [0.93–2.06]; speech/language delay: aHR = 1.33 [1.06–1.67]). Risk also differed by trimester of maternal SARS-CoV-2 infection, with over 2-fold increased risks observed with exposure in the first trimester (ND: aHR = 2.12 [1.08–4.15]; speech/language delay: aHR = 2.16 [1.06–4.47]) and second trimester (speech/language delay: aHR = 2.22 [1.23–4.00]). Results were similar for the SARS-CoV-2 spike, RBD and NTD proteins. Few of the neonates had detectable levels of IgM antibodies to the SARS-CoV-2 proteins. In conclusion, higher levels of neonatal IgG antibodies to SARS-CoV-2 proteins following infection in pregnancy may indicate increased risk for ND. If replicated, these findings suggest that newborn antibody testing of infants with prenatal SARS-CoV-2 exposure may provide a novel avenue for early identification of infants at risk for ND and opportunities for early intervention and prevention of adverse child outcomes.

## 1. Introduction

Pregnancy is associated with altered immunity and increased risk of infections. Gestational exposure to infection has been linked with adverse child outcomes, including neurodevelopmental disorders (ND). (Croen et al., 2019) Infections with severe acute respiratory syndrome coronavirus 2 (SARS-CoV-2) during pregnancy have been associated with severe maternal morbidity and preterm birth (Ferrara et al., 2022). Early studies that examined the impact of gestational exposure to SARS-CoV-2 on child neurodevelopment provided mixed results when children were followed up to 24 months of age (Pacurar et al., 2025). However, two recent large-scale studies, with longer follow-up, have found associations between SARS-CoV-2 infection and increased risk of neurodevelopmental disorders at age 3, and elevated autism risk among girls by age 4 (Croen et al., 2026; Shook et al., 2025).

Maternal immune activation (MIA) is one hypothesized mechanism by which prenatal exposure to infection can adversely impact fetal brain development (Han et al., 2021). Under normal conditions, the maternal immune system maintains a pathogen-free and non-inflammatory environment for the developing fetus (Chaouat, 2007; Wegmann et al., 1993). However, factors including cytokines and antibodies produced during gestation in response to infection can access the fetal compartment and have adverse developmental consequences (Ashdown et al., 2006; Samuelsson et al., 2006; Zaretsky et al., 2004; Hauguel-de Mouzon and Guerre-Millo, 2006). In most infected individuals, neutralizing antibodies against the SARS-CoV-2 spike protein are detectable within days to weeks of symptom development (Kirkcaldy et al., 2020; Zhao et al., 2020; Wolfel et al., 2020; To et al., 2020). In pregnancy, symptomatic individuals have higher antibody titers (IgG, IgM, and IgA) than asymptomatic, (Martin-Vicente et al., 2023) and higher antibody titers have been associated with greater clinical severity.^14^ Levels of neutralizing SARS-CoV-2 IgG antibodies generated in the beginning of pregnancy often remain elevated at later stages(Cosma et al., 2021) and are transferred across the placenta to the newborn after symptomatic as well as asymptomatic infection (Kugelman et al., 2022). Among 585 mother-child dyads with detectable maternal IgG to SARS-CoV-2 following immunization, SARS-CoV-2 IgG was detected in cord blood from 557 newborns (95.2%).(Kugelman et al., 2022) SARS-CoV-2 IgG antibody concentrations measured in umbilical cord blood generally correlate with maternal antibody concentrations and with duration between onset of infection and delivery (Flannery et al., 2021). SARS-CoV-2 IgG antibodies measured in newborn bloodspots thus reflect past or recent maternal infection. The measurement of IgM antibodies in the newborn can provide information relating to fetal infection since IgM antibodies do not generally pass through the placenta and thus arise from interactions between antigens and the neonatal immune system. As such, newborn bloodspots are an ideal sample with which to investigate the impact of MIA on child neurodevelopment in the context of gestational exposure to SARS-CoV-2.

Using newborn bloodspots, we investigated the association between neonatal levels of IgG and IgM antibodies to SARS-CoV-2 and ND among children with gestational SARS-CoV-2 exposure. We hypothesized that higher levels of IgG antibodies to SARS-CoV-2 would be associated with increased risk for ND. We also postulated that the analysis of antibodies measured in neonatal blood samples would contribute new insights into the mechanisms by which maternal infection and antibody response to SARS-CoV-2 may affect neurodevelopmental outcomes.

## 2. Materials and Methods

### 2.1. Participants

The study population for this longitudinal prospective cohort study was selected from the 4.5 million members of Kaiser Permanente Northern California (KPNC), an integrated health care system serving the San Franciso and Sacramento metropolitan areas and surrounding counties (Gordon, 2015). All KPNC members who were in any trimester of pregnancy in 2020 were invited to complete the KPNC COVID-19 Pregnancy Survey(Ames et al., 2021) which collected information regarding their experiences during the SARS-CoV-2 pandemic. Individuals meeting all of the following criteria were eligible for inclusion: 1) completed the KPNC COVID-19 Pregnancy Survey, 2) had a positive SARS-CoV-2 polymerase chain reaction (PCR) test during pregnancy, and 3) delivered a singleton livebirth (N=465). Those who received a SARS-CoV-2 vaccination prior to delivery were excluded (N=66), for a final study population of 399 mother-child dyads. This data-only study was approved by the KPNC and State of California Institutional Review Boards, which waived the requirement for informed consent from participants, as well as by the Institutional Review Board of the Johns Hopkins School of Medicine. The study followed the Strengthening the Reporting of Observational Studies in Epidemiology (STROBE) reporting guidelines.

### 2.2. Neonatal SARS-CoV-2 Antibody measurement

Newborn screening bloodspots were retrieved from the California Biobank Program of the California Department of Public Health. Specimens were collected by the heel-stick method within 48–72 hours of birth, spotted on Schleicher & Schuell filter paper dried at room temperature prior to transport to the regional laboratory. Following newborn metabolic screening, residual specimens were stored at −15°F.

Prior to testing, blood was eluted from one 10mm dried blood spot by incubation with 100 ul phosphate buffered saline containing 0.01 % Tween 20 for 1 hour at 37 degrees. We measured IgG class antibodies to recombinant SARS-CoV-2 nucleocapsid as well as to three spike antigens: the full-length spike protein, the s1-receptor binding domain (RBD), and the s1 N-terminal domain (NTD) of the spike protein employing multiplex chemiluminescence assays as previously described (Reagents obtained from Mesoscale Discovery, Rockville, Maryland USA) (Dickerson et al., 2015). A composite SARS-CoV-2 spike IgG antibody measure was calculated from principal components analysis based on the highly intercorrelated levels of antibodies to the three spike antigens following procedures which have been previously described (McNaughton et al., 2022). We also employed similar chemiluminescence assays to measure IgM class antibodies to the SARS-CoV-2 nucleocapsid protein as well as to the SARS-CoV-2 spike and receptor binding domain (RBD) proteins.

### 2.3. Neurodevelopmental disorders

All children with ND were identified using International Classification of Diseases 10^th^ Revision (Organization, 2004) diagnoses recorded in their electronic health record from 3 months after birth through June 30, 2025: intellectual disability (F70-F79), speech/language delay (F80), learning disorder (F81), motor delay (F82), ASD (F84.0, F84.5, F84.8, F84.9), and other developmental disorders (F84.2, F84.3, F88, F89). KPNC implements a universal child developmental screening program at the 18- and 24-month well-child visit with secondary screening followed by standardized assessment by autism experts for ASD concerns. Children were followed up to 64 months with a median of 48 months.

### 2.4. Covariates

Maternal and infant characteristics associated with COVID-19 infection in pregnancy or ND risk in this or previous studies were retrieved from the maternal and child inpatient and outpatient electronic health records. These included maternal age, race/ethnicity, education, insurance type, season of delivery, parity, pre-pregnancy obesity, and child gestational age, birthweight, and sex. There was no missing data for any of these covariates.

### 2.5. Statistical analysis

Levels of SARS-CoV-2 IgG antibody directed at SARS-CoV-2 nucleocapsid and spike proteins were natural log-transformed and distributions were plotted and compared by trimester of infection, child sex, and pre-pregnancy BMI with ANOVA or t-tests. We ran separate Cox proportional hazards regression models to estimate hazard ratios (HR) and 95% confidence intervals (CIs) for each child outcome with sufficient sample size (all ND combined, ASD, speech/language delay, and motor delay), associated with each neonatal IgG. Children were followed from 3 months of age until the date of the first ND diagnosis, the date they left the KPNC health system, death, or June 30, 2025, whichever occurred first. Multivariate models included adjustment for maternal age, pre-pregnancy obesity, season of childbirth, trimester of maternal COVID-19 infection, and child sex. We also conducted stratified analyses to examine differences in association by child sex or trimester of infection and then included interaction terms to test for statistical significance of differences in associations by subgroup characteristics. Additional Cox models with restricted cubic splines with reference at 10% were conducted to visualize the shape of the associations between neonatal antibody levels and overall ND risk stratified by child sex.

We analyzed the SARS-CoV-2 IgM levels with a focus on identifying neonates with levels of these antibodies greater than background. This was accomplished by defining a cutoff based on the levels of presumed negative samples defined as those of infants who did not have detectable SARS-CoV-2 IgG levels in their neonatal blood samples and who were born to mothers who did not have SARS-CoV-2 infection during pregnancy. A neonatal blood spot sample was considered to contain detectable levels of SARS-CoV-2 IgM if it had a value more than 2 standard deviations greater than the mean of the presumed negative samples.

## 3. Results

Among the 399 children (208 females,191 males) with gestational exposure to maternal SARS-CoV-2 infection, 95% had gestational age ≥37 weeks and birthweight ≥2500 grams. The mean maternal age was 31 +− 5 years; 12% were Asian, 5% Black, 45% Hispanic, 33% White, and 5% other/unknown race/ethnicity; 80% had more than a High School education; 86% had commercial insurance; 37% were primiparous; and 35% had pre-pregnancy obesity (Table 1).

A total of 73 children were diagnosed with any ND in follow-up (22 ASD, 67 speech/language disorder, 11 motor delay). Baseline characteristics of children diagnosed with ND were similar to children without ND with respect to maternal age, race/ethnicity, education, insurance type, season of birth, parity, maternal pre-pregnancy obesity, and child gestational age and birthweight. Among children diagnosed with ND in follow-up, 64.4% were male (Table 1).

**Fig. 1.**
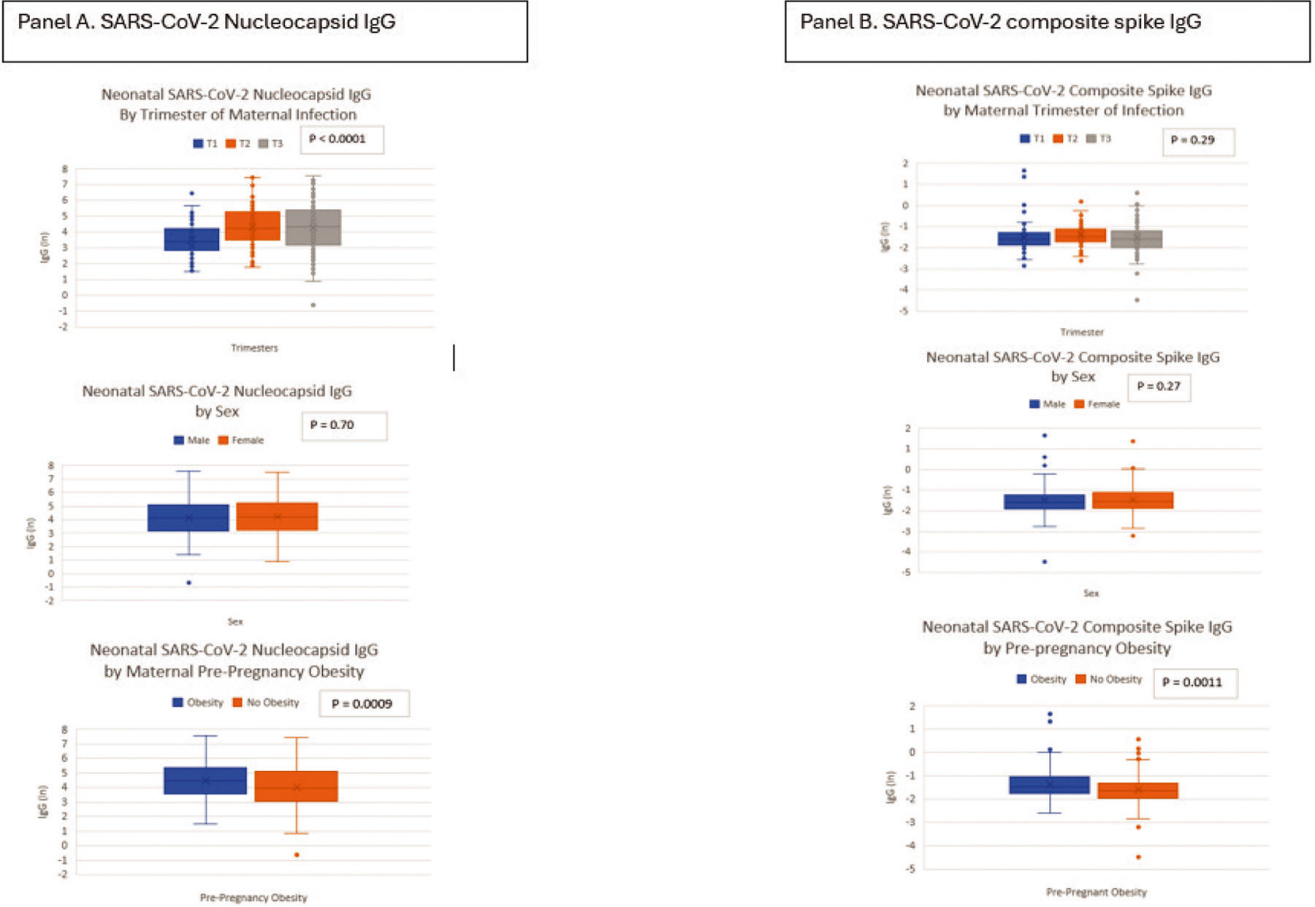
Distribution of SARS-CoV-2 nucleocapsid and spike IgG antibodies measured in newborn bloodspots of children whose mothers had confirmed SARS-CoV-2 infection during pregnancy.

Fig. 1 shows the distribution of natural log-transformed SARS-CoV-2 IgG antibodies measured in newborn bloodspots of children whose mothers had confirmed COVID-19 infection during pregnancy. Levels of neonatal SARS-CoV-2 nucleocapsid IgG were significantly higher among the children with maternal pre-pregnancy obesity and SARS-CoV-2 infection in the second and third trimester of pregnancy (Fig. 1A). Levels of the SARS-CoV-2 composite spike IgG antibody were also significantly higher among children with maternal pre-pregnancy obesity (Fig. 1B).

**Fig. 2.**
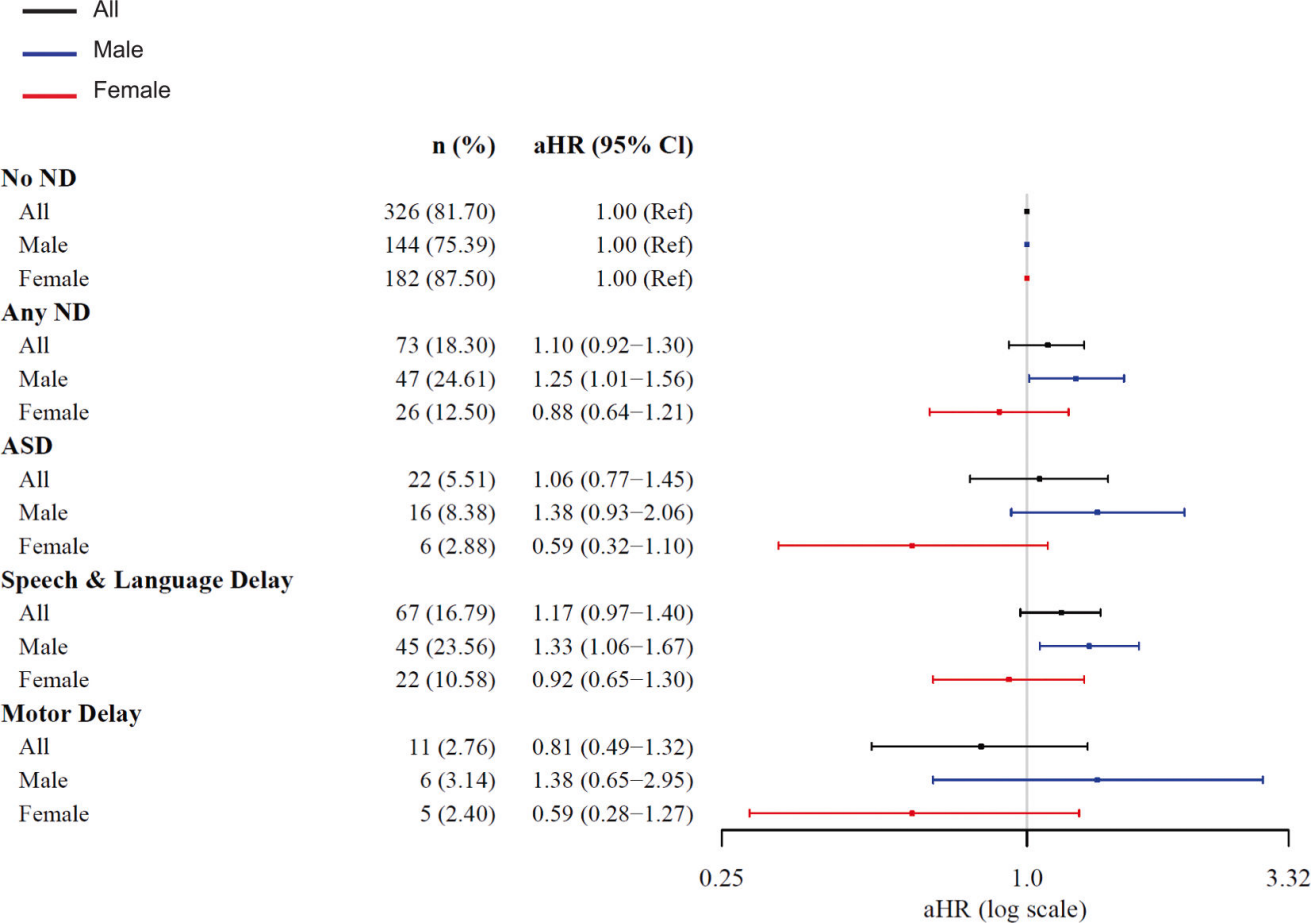
SARS-CoV-2 Nucleocapsid IgG by Sex. Abbreviations: adjusted hazard ration (aHR); Autism Spectrum Disorder (ASD); confidence interval (CI); Immunoglobulin G (IgG); neurodevelopmental disorders (ND).

**Fig. 3.**
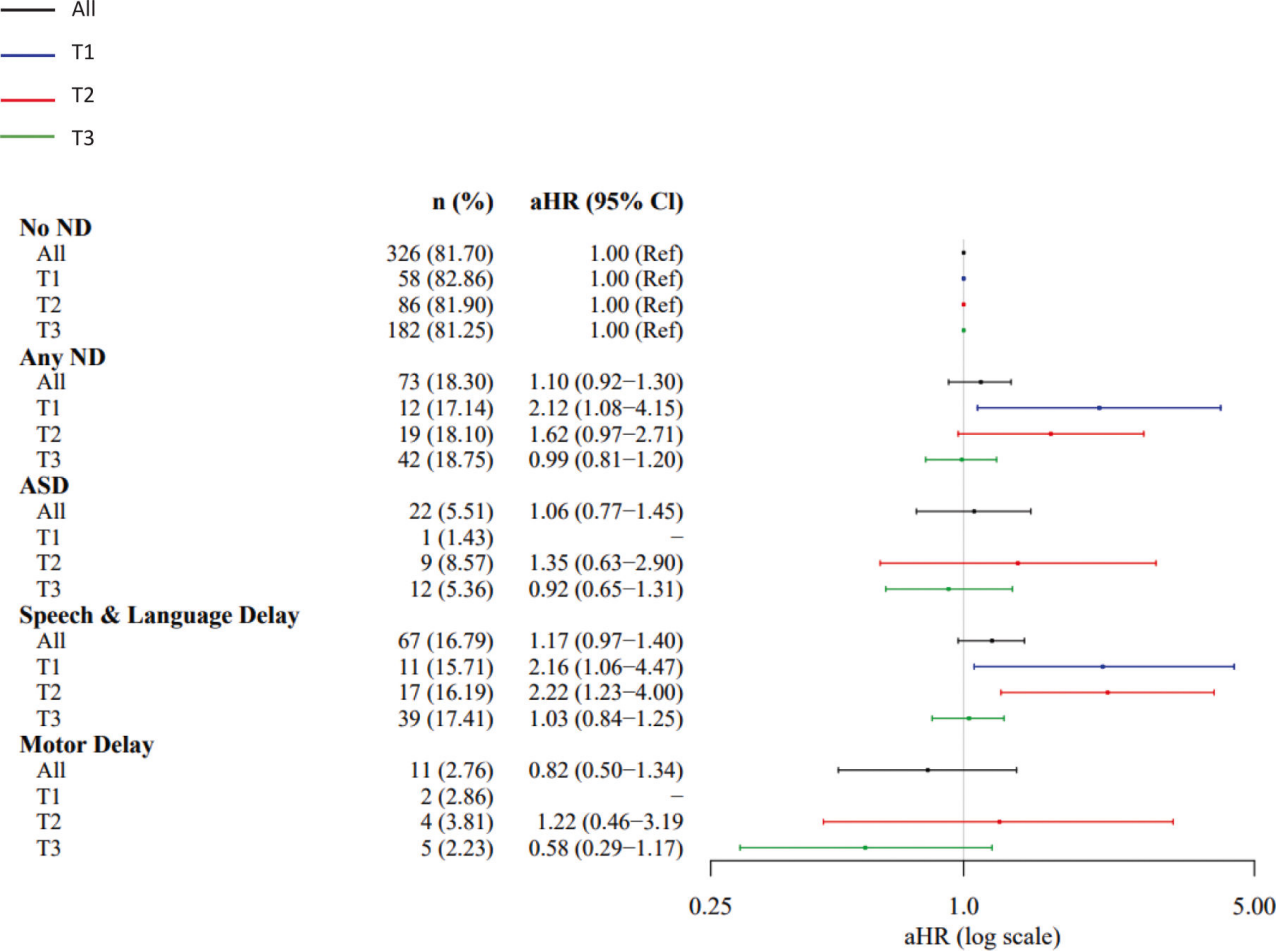
SARS-CoV-2 Nucleocapsid IgG by trimester of infection. Abbreviations: adjusted hazard ration (aHR); Autism Spectrum Disorder (ASD); confidence interval (CI); Immunoglobulin G (IgG); neurodevelopmental disorders (ND); Trimester (T).

**Fig. 4.**
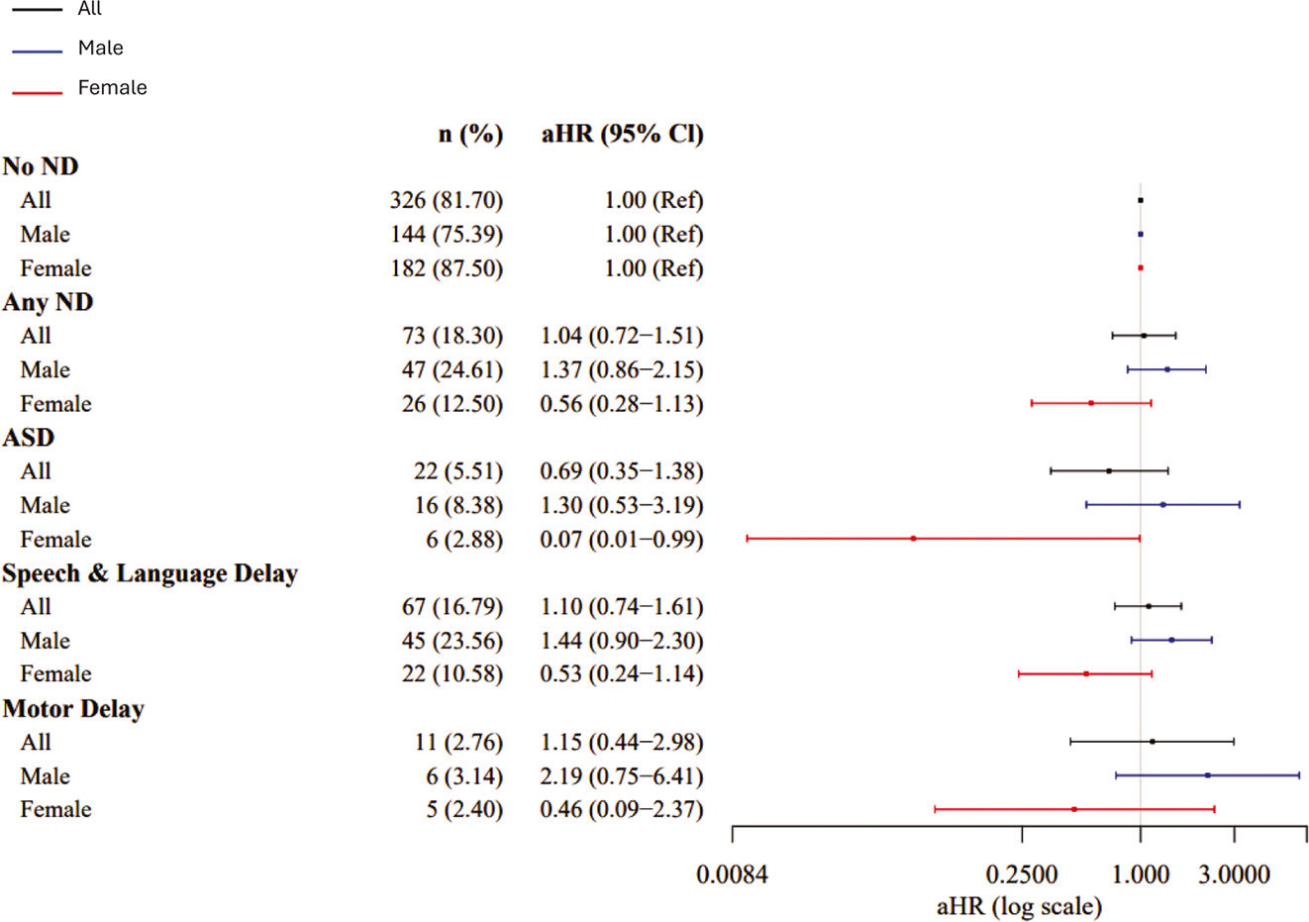
SARS-CoV-2 Composite Spike IgG by sex. Abbreviations: adjusted hazard ration (aHR); Autism Spectrum Disorder (ASD); confidence interval (CI); Immunoglobulin G (IgG); neurodevelopmental disorders (ND).

**Fig. 5.**
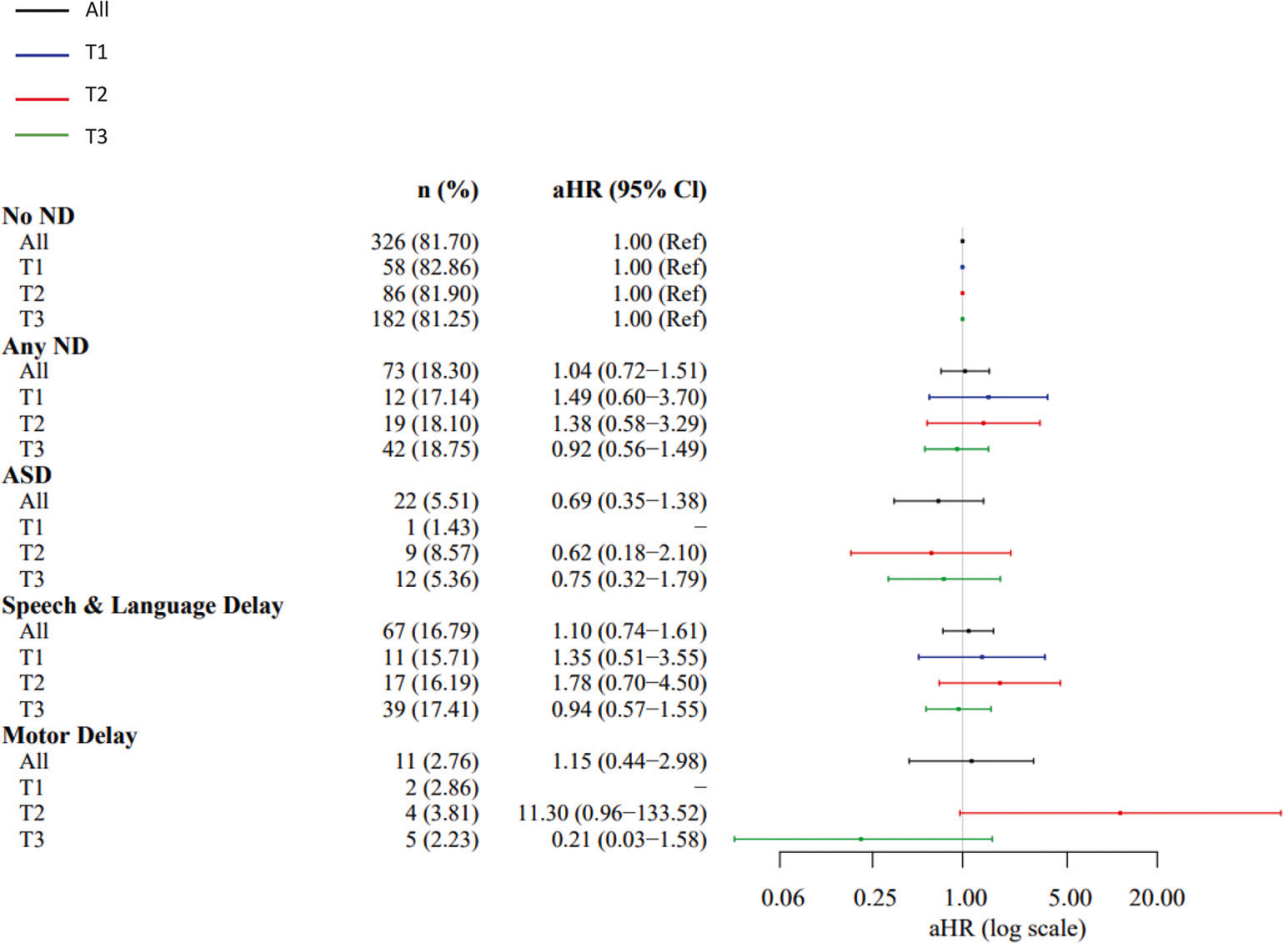
SARS-CoV-2 Composite Spike IgG by trimester of infection. Abbreviations: adjusted hazard ration (aHR); Autism Spectrum Disorder (ASD); confidence interval (CI); Immunoglobulin G (IgG); neurodevelopmental disorders (ND); Trimester (T).

**Fig. 6.**
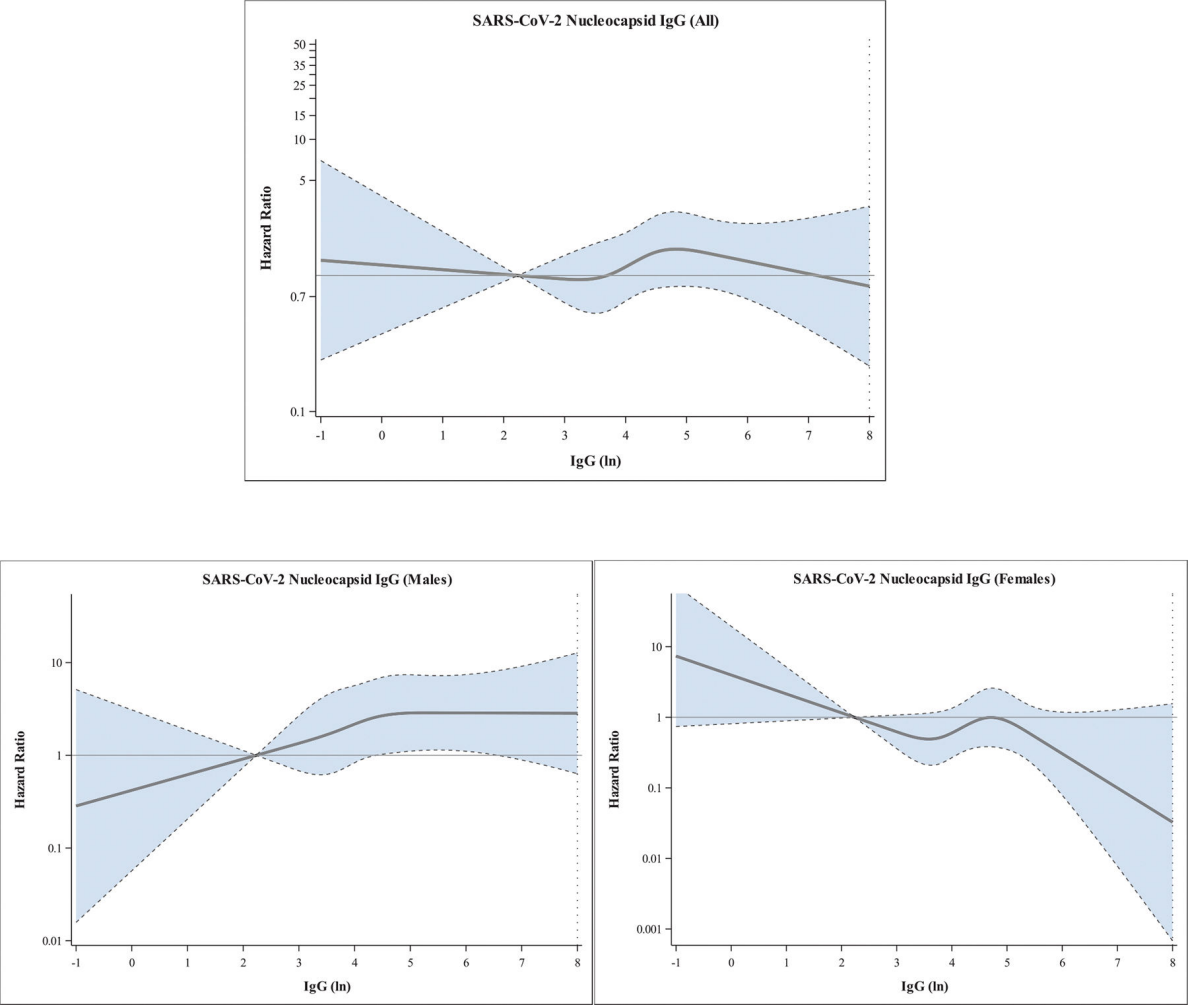
Restricted cubic spline Cox regression for risk of ND associated with newborn levels of SARS-CoV-2 nucleocapsid IgG antibody in newborn bloodspots. Top – all children; Bottom left – Males; Bottom right – Females. Fig. 6B: Restricted cubic spline Cox regression for risk of ND associated with newborn levels of SARS-CoV-2 Composite Spike IgG antibody in newborn bloodspots. Top – all children; Bottom left – Males; Bottom right – Females.

SARS-CoV-2 nucleocapsid IgG antibody level was not associated with increased risk of ND among the overall sample (aHR=1.10, 95% CI [0.92–1.30]), ASD (aHR=1.06 [0.77–1.45]), speech/language delay (aHR=1.17 [0.97–1.40]), or motor delay (aHR=0.82 [0.50–1.34]) in analyses adjusted for maternal age, pre-pregnancy obesity, child birth season, maternal Covid-19 infection trimester, and child sex (Table 2a, Fig. 2). However, associations differed significantly by child sex, with increased risk observed for each outcome only among males (ND: aHR=1.25 [1.01–1.56]; ASD: aHR=1.38 [0.93–2.06]; Speech/Language Delay: aHR=1.33 [1.06–1.67]; Motor Delay: aHR=1.38 [0.65–2.95] (Table 2a, Fig. 2). Risk also differed by trimester of maternal SARS-CoV-2 infection, with over 2-fold increased risks observed among the overall sample with first (ND: aHR=2.12 [1.08–4.15]; Speech/Language: aHR=2.16 [1.06–4.47]) and second (Speech/Language: aHR=2.22 [1.33–4.00]) trimester exposure (Table 2a, Fig. 3). The direction and magnitude of results was similar for the IgG antibodies directed at SARS-CoV-2 composite spike proteins (Table 2b, Figs. 4 and 5). Results of restricted cubic spline Cox regression models for all ND combined associated with neonatal SARS-CoV-2 nucleocapsid and the composite spike IgG antibody level were consistent with our main findings and showed no evidence of non-linearity in associations (Fig. 6).

We also measured IgM class antibodies to the SARS-CoV-2 nucleocapsid, spike, and RBD proteins. There were only 9 neonatal samples with detectable levels of IgM antibodies to the nucleocapsid protein, 10 with detectable levels of IgM to the RBD protein, and none to the spike protein. Only one of these samples was from an infant who was subsequently diagnosed with ND.

## 4. Discussion

Among a large and diverse population of children with prenatal exposure to SARS-CoV-2 infection followed up to 64 months of age, higher neonatal SARS-CoV-2 IgG antibody levels were associated with increased risk of ND among males, regardless of trimester of SARS-CoV-2 exposure, and among children of both sexes with SARS-CoV-2 exposure in early gestation. The mothers had not received any of the SARS-CoV-2 vaccines, hence the SARS-CoV-2 IgG antibody levels could not be ascribed to immunization. In contrast to the associations with IgG class antibodies, there was little evidence of the generation of IgM antibodies to SARS-CoV-2 proteins in the offspring of infected mothers. Since maternal IgM antibodies do not readily cross the placenta, IgM class antibodies in neonatal blood samples are generally a sign of prenatal infection of the fetus. These antibodies are commonly found in prenatal infections known to involve cross-placental infections such as those caused by cytomegalovirus, rubella virus and Toxoplasma gondii (Hirota et al., 1992; Kaneko et al., 2006; Marangoni et al., 2014). While some false negative IgM levels are possible, the virtual absence of these antibodies suggest that perinatal SARS-CoV-2 infections measured by the parameters applied to other pathogens were uncommon in our population. Our findings suggest that the effect of SARS-CoV-2 on the mother rather than direct in utero infection of the offspring is associated with increased risk of ND.

There are several possible mechanisms by which maternal infection can affect the offspring in the absence of direct prenatal infection. One of the best documented mechanisms involves MIA where the generation of cytokines and other inflammatory markers affect prenatal brain development. This process has been identified in rodent and primate models and is consistent with human studies demonstrating associations with neurodevelopmental outcomes, for example between markers of maternal systemic immune activation and increased risk of ASD (Han et al., 2021; Marangoni et al., 2014; Zawadzka et al., 2021). In the case of SARS-CoV-2 our group has demonstrated an association between prenatal SARS-CoV-2 infection, levels of neonatal cytokines and chemokines and subsequent risk of ND (Kim et al., 2025).

We found that increased levels of SARS-CoV-2 IgG antibodies were associated with increased risk of ND in male children only. Consistent with these sex-specific findings, a study of over 18,000 births in Massachusetts found that PCR evidence of SARS-CoV-2 infection during pregnancy was associated with an elevated risk of neurodevelopmental diagnoses in the first years of life among male offspring.^5^ Similarly, studies on neonates and infants following post-natal infections have generally found more serious consequences for males than females (Muenchhoff and Goulder, 2014; Elsmén et al., 2004).

In the absence of apparent infection, pregnant individuals carrying a female fetus generally exhibit greater stimulated cytokine production throughout pregnancy compared to those carrying a male fetus (Mitchell et al., 2017). Under normal conditions, cytokines mediate signals between the immune and nervous systems that help shape neuronal responses, neuronal circuit development, early brain growth, and subsequent behaviors. When such homeostatic conditions are perturbed by infection, a shift in the maternal response could impact the offspring. Murine models of lipopolysaccharide-induced MIA during pregnancy demonstrate distinct sex-specific developmental consequences, with males experiencing more pronounced social and learning-behavioral abnormalities (Braun et al., 2019). These are likely due to sex-biased activation of the integrated stress response (ISR) where the brains of male MIA-exposed offspring exhibit a sexually dimorphic ISR that is characterized by activation of PERK and eIF2α signaling, both of which are hallmarks of endoplasm reticulum stress and lead to an attenuation of protein synthesis (Kalish et al., 2021). Convergent evidence from animal and human studies demonstrates significant sex differences in the placental response to maternal immune challenges which may, in turn, lead to sex differences in the fetal brain (Guma and Chakravarty, 2025).

With respect to the timing of maternal infection we found that SARS-CoV-2 infection during the first trimester conferred the highest risk of adverse neurodevelopmental outcomes in both sexes combined. This finding is consistent with the high degree of fetal brain development which occurs early in pregnancy and the susceptibility during this period to the effects of a range of environmental factors including infection (Stolp et al., 2012; Molnar and Kennedy, 2016). In terms of SARS-CoV-2 this finding is consistent with the increased rates of preterm birth and placental pathology following SARS-CoV-2 early in pregnancy (Dagelic et al., 2023; Seif et al., 2023). The possibility that the increased risk early in pregnancy is related to MIA is supported by a study of gene expression in placental cells obtained from women with confirmed SARS-CoV-2 infection at different stages of pregnancy (Xi et al., 2024). That study documented increased transcription of RNA coding immune factors during the first trimester with levels decreasing during later stages of pregnancy. Since IgG antibodies in the neonatal blood samples derive from the mother, our finding that the levels of IgG antibodies to SARS-CoV-2 measured in the neonatal blood samples were lowest following maternal infection in the first trimester may reflect decreasing levels of SARS-CoV-2 IgG antibodies during pregnancy, as has been noted following SARS-CoV-2 immunization (Zilver et al., 2023; Atyeo et al., 2022). It is also possible that the efficiency of the transfer of antibodies from the mother to the neonate was impacted by the effects of infection on the placenta as has been previously reported (Coler et al., 2024; Okoeguale et al., 2023). IgG levels could represent the degree of viral replication in infected individuals as well as the extent of the maternal immune response. Thus, increased maternal levels may represent a more severe maternal infection as well as maternal immune dysfunction in the presence of virus.Table 2b

SARS-CoV-2 Composite spike IgG in newborn bloodspots of children whose mothers had confirmed COVID-19 during pregnancy.

### 4.1. Strengths and Limitations

This is the largest and most comprehensive study to date examining the neonatal immune profile in relation to subsequent diagnoses of neurodevelopmental disorders among a cohort of children prenatally exposed to PCR-confirmed SARS-CoV-2 infection. After birth, children were followed for up to 5 years, longer than previous studies, allowing for more comprehensive ascertainment of physician diagnosed ND outcomes which were prospectively recorded in children’s medical records. Our analyses excluded individuals who had ever received a SARS-CoV-2 vaccination and analyses adjusted for several potential confounding factors. This, together with the measurement of both IgG and IgM antibodies to multiple SARS-CoV-2 proteins allowed us to distinguish the impact of maternal infection during pregnancy from prenatal infection of the neonate.

One major limitation of the study was the lack of availability of maternal prenatal blood samples and fetal brain tissue, and thus the inability to directly measure the maternal immune response to SARS-CoV-2 infection and the role of antibody-dependent enhancement (ADE) in fetal brain. Second, while we measured antibodies to a number of viral proteins which have been shown to be correlated with neutralization, (McConnell et al., 2023) we did not directly measure neutralization or other functional activities. Third, lack of adjustment for other factors associated with maternal SARS-CoV-2 infection severity and ND risk, including maternal symptom severity, medication use during pregnancy, concurrent infections, stress, and environmental exposures may have confounded our results. Fourth, our analyses did not account for the potential impact of variability in maternal immune-related genes. Future large studies with collection of multiple samples throughout pregnancy, long-term follow-up of children, and consideration of gene-environment interactions are needed.

## 5. Conclusion

Higher levels of neonatal IgG antibodies following in utero exposure to maternal SARS-CoV-2 infection may indicate increased risk for ND, especially among males and following first trimester infections. If replicated in an expanded sample, enabling the definition of a clinically meaningful threshold, these findings suggest that newborn antibody testing of male infants with prenatal SARS-CoV-2 exposure can provide a novel avenue for early identification of infants at risk of ND and opportunities for early intervention and prevention of adverse child outcomes. Our findings also support the promotion of SARS-CoV-2 immunization prior to the start of pregnancy to prevent infections in the first trimester as well as the treatment of mothers infected early in pregnancy to limit viral replication and immune activation.

## Figures and Tables

**Table 1:** Characteristics of study population by child ND status, children with prenatal exposure to SARS-CoV-2 infection born April 2020-September 2021 at Kaiser Permanente Northern California.

Characteristic	All (N = 399) N (%)	ND (N = 73) N (%)	No ND (N = 326) N (%)	P-value

**Trimester COVID-19**				
T1	70 (17.54)	12 (16.44)	58 (17.79)	0.9531
T2	105 (26.32)	19 (26.03)	86 (26.38)	
T3	224 (56.14)	42 (57.53)	182 (55.83)	
**Maternal age, Mean (SD)**	30.70 (5.17)	30.68 (5.42)	30.70 (5.12)	0.9792
**Maternal Race**				
White	131 (32.83)	23 (31.51)	108 (33.13)	0.8278
Black	22 (5.51)	3 (4.11)	19 (5.83)	
Asian	47 (11.78)	9 (12.33)	38 (11.66)	
Hispanic	179 (44.86)	36 (49.32)	143 (43.87)	
Other	15 (3.76)	2 (2.74)	13 (3.99)	
Unknown	5 (1.25)	0 (0.00)	5 (1.53)	
**Maternal Education**				
Less High School	3 (0.75)	1 (1.37)	2 (0.61)	0.7694
High School	76 (19.05)	15 (20.55)	61 (18.71)	
College	244 (61.15)	44 (60.27)	200 (61.35)	
Post-Graduate	42 (10.53)	9 (12.33)	33 (10.12)	
Unknown	34 (8.52)	4 (5.48)	30 (9.20)	
**Maternal Insurance Type**				
Commercial	345 (86.47)	62 (84.93)	283 (86.81)	0.4254
Government	49 (12.28)	11 (15.07)	38 (11.66)	
Unknown	5 (1.25)	0 (0.00)	5 (1.53)	
**Season of Delivery**				
Spring 2020	8 (2.01)	3 (4.11)	5 (1.53)	0.4314
Summer 2020	42 (10.53)	10 (13.70)	32 (9.82)	
Fall 2020	73 (18.30)	14 (19.18)	59 (18.10)	
Winter 2021	128 (32.08)	25 (34.25)	103 (31.60)	
Spring 2021	85 (21.30)	11 (15.07)	74 (22.70)	
Summer 2021	63 (15.79)	10 (13.70)	53 (16.26)	
**Parity**				
0	149 (37.34)	35 (47.95)	114 (34.97)	0.4224
1	129 (32.33)	20 (27.40)	109 (33.44)	
2	75 (18.80)	12 (16.44)	63 (19.33)	
3	22 (5.51)	2 (2.74)	20 (6.13)	
4+	11 (2.76)	2 (2.74)	9 (2.78)	
Unknown	13 (3.26)	2 (2.74)	11 (3.37)	
**Pre-pregnancy obesity**	138 (34.59)	26 (35.62)	112 (34.36)	0.8378
**Gestational Age**				
<35 weeks (early preterm)	6 (1.50)	2 (2.74)	4 (1.23)	0.5971
35–<37 weeks (preterm)	14 (3.51)	3 (4.11)	11 (3.37)	
≥37 weeks (term)	379 (94.99)	68 (93.15)	311 (95.40)	
**Birthweight**				
<1500gm (very low bwt)	2 (0.50)	1 (1.37)	1 (0.31)	0.4827
1500–2499gm (low bwt)	14 (3.51)	3 (4.11)	11 (3.37)	
2500 + gm (normal bwt)	383 (95.99)	69 (94.52)	314 (96.32)	
**Child Sex**				
Female	208 (52.13)	26 (35.62)	182 (55.83)	0.0018
Male	191 (47.87)	47 (64.38)	144 (44.17)	

Abbreviations: Birthweight (bwt); Neurodevelopmental Disorder (ND); Severe acute respiratory syndrome coronavirus 2 (SARS-CoV-2); Standard Deviation (SD).

**Table 2a:** SARS-CoV-2 Nucleocapsid IgG in newborn bloodspots of children whose mothers had confirmed COVID-19 during pregnancy.

Child Outcome	All HR^1^ (CI)		Female HR^2^ (CI)		Male HR^2^ (CI)		Sex interaction p-value	T1 HR^3^ (CI)		T2 HR^3^ (CI)		T3 HR^3^ (CI)		Trimester interaction p-value
Any ND	73	1.10 (0.92–1.30)	26	0.88 (0.64–1.21)	47	**1.25 (1.01–1.56)**	**0.0134**	12	**2.12 (1.08–4.15)**	19	1.62 (0.97–2.71)	42	0.99 (0.81–1.20)	01,462

ASD	22	1.06 (0.77–1.45)	6	0.59 (0.32–1.10)	16	1.38 (0.93–2.06)	**0.0328**	1	0.69 (0.00-.)	9	1.35 (0.63–2.90)	12	0.92 (0.65–1.31)	0.4387
Speech/Language Delay	67	1.17 (0.97–1.40)	22	0.92 (0.65–1.30)	45	**1.33 (1.06–1.67)**	**0.0193**	11	**2.16 (1.06–4.47)**	17	**2.22 (1.23–4.00)**	39	1.03 (0.84–1.25)	0.0481
Motor delay	11	0.82 (0.50–1.34)	5	0.59 (0.28–1.27)	6	1.38 (0.65–2.95)	0.0990	2	–	4	1.22 (0.46–3.19)	5	0.58 (0.29–1.17)	0.3102

1 Adjusted by maternal age, maternal pre-pregnancy obesity, child birth season, maternal covid infection trimester, and child sex.

2 Adjusted by maternal age, maternal pre-pregnancy obesity, child birth season, and maternal covid infection trimester.

3 Adjusted by maternal age, maternal pre-pregnancy obesity, child birth season, and child sex.

Abbreviations: Autism Spectrum Disorder (ASD); Confidence interval (CI); Hazard ratio (HR); Immunoglobulin G (IgG); Neurodevelopmental Disorder (ND); Severe acute respiratory syndrome coronavirus 2 (SARS-CoV-2); Trimester 1 (T1); Trimester 2 (T2); Trimester 3 (T3).

**Table 2b:** SARS-CoV-2 Composite spike IgG in newborn bloodspots of children whose mothers had confirmed COVID-19 during pregnancy.

Child Outcome	All HR^1^ (CI)		Female HR^2^ (CI)		Male HR^2^ (CI)		Sex interaction p-value	T1 HR^3^ (CI)		T2 HR^3^ (CI)		T3 HR^3^ (CI)		Trimester interaction p-value
Any ND	73	1.04 (0.72–1.51)	26	0.56 (0.28–1.13)	47	1.37 (0.86–2.15)	**0.0110**	12	1.49 (0.60–3.70)	19	1.38 (0.58–3.29)	42	0.92 (0.56–1.49)	0.8138

ASD	22	0.69 (0.35–1.38)	6	**0.07 (0.01–0.99)**	16	1.30 (0.53–3.19)	**0.0143**	1	0.29 (0.00-.)	9	0.62 (0.18–2.10)	12	0.75 (0.32–1.79)	0.8845
Speech/Language Delay	67	1.10 (0.74–1.61)	22	0.53 (0.24–1.14)	45	1.44 (0.90–2.30)	**0.0107**	11	1.35 (0.51–3.55)	17	1.78 (0.70–4.50)	39	0.94 (0.57–1.55)	0.6307
Motor delay	11	1.15 (0.44–2.98)	5	0.46 (0.09–2.37)	6	2.19 (0.75–6.41)	0.0602	2	–	4	11.30 (0.96–133.52)	5	0.21 (0.03–1.58)	0.0476

1 Adjusted by maternal age, maternal pre-pregnancy obesity, child birth season, maternal covid infection trimester, and child sex.

2 Adjusted by maternal age, maternal pre-pregnancy obesity, child birth season, and maternal covid infection trimester.

3 Adjusted by maternal age, maternal pre-pregnancy obesity, child birth season, and child sex.

Abbreviations: Autism Spectrum Disorder (ASD); Confidence interval (CI); Hazard ratio (HR); Immunoglobulin G (IgG); Neurodevelopmental Disorder (ND); Severe acute respiratory syndrome coronavirus 2 (SARS-CoV-2); Trimester 1 (T1); Trimester 2 (T2); Trimester 3 (T3).

## Data Availability

Data will be made available on request.

## Abbreviations:

Cis: Confidence intervals
HR: Hazard ratios
IgG: Immunoglobulin G
IgM: Immunoglobulin M
IgA: Immunoglobulin A
KPNC: Kaiser Permanente Northern California
MIA: Maternal immune activation
ND: Neurodevelopmental disorders
NTD: N-terminal domain
SARS-CoV-2: Severe acute respiratory syndrome coronavirus 2
STROBE: Strengthening the Reporting of Observational Studies in Epidemiology
PCR: Polymerase chain reaction
RBD: Receptor binding domain
